# Polymorphism of DNA Methyltransferase 3b and Association with Development and Prognosis in Gastric Cancer

**DOI:** 10.1371/journal.pone.0134059

**Published:** 2015-08-11

**Authors:** Chuan Wang, Zhifang Jia, Donghui Cao, Lili You, Meishan Jin, Xing Wu, Simin Wen, Xueyuan Cao, Jing Jiang

**Affiliations:** 1 Division of Clinical Epidemiology, First Hospital of Jilin University, Changchun, Jilin, 130021, China; 2 Division of Pathology, First Hospital of Jilin University, Changchun, Jilin, 130021, China; 3 Department of Gastric and Colorectal Surgery, First Hospital of Jilin University, Changchun, Jilin, 130021, China; Centro di Riferimento Oncologico, IRCCS National Cancer Institute, ITALY

## Abstract

**Objective:**

DNA methyltransferase 3b (DNMT3b) plays an important role in abnormal methylation during tumorigenesis. Polymorphism of the *DNMT3b* gene may influence *DNMT3b* activity and be associated with cancer risk. This study aimed to investigate the association between single nucleotide polymorphisms (SNPs) of the *DNMT3b* gene and susceptibility and prognosis of gastric cancer.

**Methods:**

Four hundred and forty-seven histologically-confirmed gastric cancer cases, 111 gastric atrophy cases and 961 tumor-free controls were enrolled into the study. Five tag SNPs (rs6119954, rs1569686, rs4911107, rs4911259 and rs8118663) of the *DNMT3b* gene were genotyped by TaqMan assay. DNMT3b expression was evaluated in 104 cancer tissues by immunohistochemistry method.

**Results:**

The median follow-up time for 422 gastric patients with prognosis information was 55.1 (51.8–58.5) month. We found that individuals with the rs1569686 variant genotype (TG/GG) were significantly associated with poor prognosis in gastric cancer compared to those carrying the TT genotype (HR = 1.43, 95%CI: 1.02–1.99). This trend was more evident in the long-term survival of gastric cancer. Similar results were observed for the G allele carriers of rs4911107 and T allele carriers of rs4911259 as these two sites were in complete linkage disequilibrium with rs1569686. The rs8118663 GG carriers tended to live shorter than AA/AG genotype (HR = 2.72, 95%CI: 1.45–5.12) in patients living longer than 2.0 years. None of the five SNPs was associated with the risks of gastric cancer or gastric atrophy. And no relationship was found between each of the five SNPs and DNMT3b expression.

**Conclusions:**

This study provides evidence that *DNMT3b* polymorphisms may predict long-term survival of gastric cancer. However, further studies are needed to reveal the underlying biological roles of *DNMT3b* polymorphism.

## Introduction

Gastric cancer (GC) is one of the most common malignant tumors in China and it is the second leading cause of cancer related death [1, 2]. *Helicobacter pylori* (*H*.*pylori*) has been classified as a group I carcinogen of gastric cancer by IARC in 1994 [3]. *H*.*pylori* is also one of the risk factors for gastric atrophy (GA), which is involved in the development of gastric cancer [4, 5]. Although half of the world’s population has *H*.*pylori* infection, only a small proportion of them progress to chronic atrophic gastritis and then gastric cancer. These suggest a possible role of host genetic factors in response to chronic *H*.*pylori* infection and gastric cancer development, subsequently [6].

DNA methylation is one of the epigenetic modulations and is important in transcription regulation and chromatin structure remodeling [7]. Aberrant DNA methylation could result in genome-wide hypomethylation and regional hypermethylation, which is identified as a possible mechanism of inactivation of tumor suppressor genes [8–10]. DNA methyltransferase-3b (DNMT3b) is a *de novo* methyltransferase and is over expressed in variety of tumors, such as lung cancer [11], breast carcinomas [12], hepatocellular carcinoma [13] and large B-cell lymphomas [14]. Our previous work showed that DNMT3b expressed significantly higher in gastric cancer tissue compared to that of the paired control samples [15]. And higher levels of DNMT3b were reported to be involved in lymph node metastasis of ovarian cancer [16] and shorter overall survival of acute myeloid leukemia [17]. These suggested the possible role of DNMT3b in the development and progression of tumors.

*DNMT3b* locates in chromosome 20q11.2 with a total size of 47kb. It was considered that single nucleotide polymorphisms (SNPs) within the promoter region of *DNMT3b* gene may modify gene expression levels [18]. Previous studies have shown that SNPs of *DNMT3b* were correlated with the susceptibility of various cancers, such as hepatocellular carcinoma [19], lung cancer [18], nasopharyngeal carcinomas [20], breast cancer [7] and gastric cancer [21], and Azad et al [22] have demonstrated that *DNMT3b* rs2424913 polymorphism was correlated with an increased hazard risk of in head and neck cancer. These SNPs were found to affect the activity of *DNMT3b* on DNA methylation by changing the level of DNMT3b, thereby modulating the susceptibility to cancer [23, 24].

However, as far as we know, no paper was available on the role of *DNMT3b* polymorphism in the prognosis of gastric cancer. In the present study, we examined the association between polymorphisms of *DNMT3b* gene and susceptibility of gastric cancer as well as gastric atrophy, and clinicopathological features and overall survival of gastric cancer in a Chinese population.

## Materials and Methods

### Ethics statement

This study was approved by the Ethics Committee of the First Hospital of Jilin University. Written informed consents were obtained from all the subjects prior to taking part in this research.

### Study populations

Newly diagnosed gastric cancer cases undergoing tumorectomy were invited to the study in the Department of Gastric and Colorectal Surgery, First Hospital of Jilin University (Changchun, China) during 2008 to 2010. A total of 447 cases with histopathologically diagnosed gastric cancer were included and none of the cases were received chemotherapy or radiotherapy prior to surgery. The principal clinical characteristics were collected from the medical records or through telephone interview. We defined chemotherapy as an effective treatment for at least 3 cycles. The patients received several postoperative chemotherapy regimens, including FOLFOX-4 regimen (combination with 5-fluorouracil, leucovorin and oxaliplatin); XELOX regimen (capecitabine and oxaliplatin); other chemotherapies such as capecitabine or 5-fluorouracil alone. During the same period, examinees attending the health check-up center without tumor history were invited to the control group in the same hospital and 1072 individuals signed the informed consent. Among them, 150 subjects were found to have gastric atrophy by serum pepsinogen examination and 111 were confirmed by biopsy and histopathology and were included as the gastric atrophy group. The remaining subjects (961) were included in the control group. Five milliliters peripheral blood was collected from all the participants and stored at -80°C until genomic DNA extraction.

Gastric cancer cases were followed-up by telephone calls three month, six month, and one year after the tumorectomy and every one year later until death or the last scheduled follow-up. Cases would not be included in the survival analysis if (i) they were lost to follow-up at the first time of telephone interview, or (ii) they were died of complications of the surgical operation in the perioperative period. The survival time was defined as the duration from the date of surgical operation to the date of death if the patients were died or to the date of the last successful interview if the patients were lost to follow-up or alive until the end of the study. Survival time was right censored except that the patients were died of gastric cancer.

### Tests of *H*.*pylori* infection and diagnosis of gastric atrophy

Serum Immunoglobulin G (IgG) antibodies to *H*. *pylori*, pepsinogen I (PGI) and pepsinogen II (PGII) were evaluated by enzyme-linked immunosorbent assay (ELISA) following the manufacturer’s instruction (Biohit, Finland). Individuals with PGI< 82.3 ng/ml and the ratios of PGI/PGII< 6.05 were positive for gastric atrophy screening and then validated by biopsy and histopathology through gastroscopy [25]. The interday coefficient of variations (CVs) of the control samples among kits were 4.5%, 4.3% and 4.7% for *H*.*pylori* IgG, PG I and PG II, respectively.

### Tagging SNPs selection

SNPs covering the region of *DNMT3b* were analyzed using SNPbrowser Software v4.0 based on the Han Chinese Population in the HapMap Project (06-02-2009 HapMap). Four tagging SNPs (tagSNPs, rs6119954, rs4911107, rs4911259 and rs8118663) were selected which could cover the 22 SNPs of *DNMT3b* with a minimum minor allele frequency (MAF) of 0.05 and a pair-wise *r* ^2^ of 0.8 or greater [26]. In addition, three other SNPs, rs2424913, rs6087990 and rs1569686, were also intended to be selected as the candidate SNPs. However, only rs1569686 were genotyped as rs2424913 had too low MAF (0.012, data from HapMap Project) and rs6087990 was in an absolute LD (*D*’ = 1 and *r* ^2^ = 1) with rs4911107. Finally, five SNPs, rs6119954 (intron), rs1569686 (promoter), rs4911107 (intron), rs4911259 (intron) and rs8118663 (3’ flanking region) were selected for genotyping.

### Genotyping

Genomic DNA was extracted from whole blood sample using blood genomic DNA extraction kits following the manufacturer’s instructions (Axygen Biosciences, USA). Genotypes of each SNP were determined using the TaqMan genotyping assays following the manufacturer’s protocol in the 384-well plates (Applied Biosystems, USA). Sequences of primers and probes are available on request. Polymerase chain reactions (PCR) were as follows: 1 cycle of 95°C for 10 min, followed by 40 cycles of 95°C for 15 s and 60°C for 1 min. The amplification processes were performed on BIO-RAD S1000 thermal cyclers (Bio-Rad, California) and the final products were read on an ABI PRISM 7900 HT Sequence Detector using the Sequence Detector Software V2.3 (Applied Biosystems, USA). Two blank controls were included in each 384-well assay facilitating the software to identify genotypes. Five percent of randomly-selected duplicate samples were included in each 384-well plates for quality control, and the overall concordant rate was 99.91%.

### Immunohistochemistry

DNMT3b expression was assessed in tumor tissue of 104 gastric cancer patients by immunohistochemistry method. The 4μm-thick sections from tissue blocks were excised, deparaffinized and stained using a streptavidin-biotin immunoperoxidase technique. Briefly, the tissue sections were incubated overnight at 4°C with anti-human DNMT3b polyclonal antibody (1:200 diluted, sc-20704, Santa Cruz, USA). Signals were visualized with 3, 3-Diaminobenzidine (DAB) and the slides were counterstained with hematoxylin. As the negative controls, the slides were treated with the isotype IgGs as replacement of primary antibodies. The stained slides were evaluated by two independent pathologists, who were blinded from clinical data. The widely accepted HSCORE system was used to assess staining intensity and percentages of the cells stained with a specific magnitude of intensity. The HSCORE was calculated by the following equation: HSCORE = ∑Pi(i) (i = 0, 1, 2, 3, Pi = 0–100%). The i means the intensity of staining, i.e. no staining = 0, weak staining = 1, moderate staining = 2 and strong staining = 3. Pi represents percentages of stained cells with intensities varying from 0 to 100%. The HSCORE ranges from 0 to 300.

### Statistical analysis

Continuous data such as age and HSCORE scores of DNMT3b expression were summarized as median (25th to 75th percentiles) and compared by Mann-Whitney U test or Kruskal-Wallis test. Categorical data were described as frequency and percentage and compared using χ^2^ test or Fisher exact test when appropriate. The frequencies of genotypes of each SNP were determined via direct counting and deviation from Hardy-Weinberg equilibrium in control group was assessed by a goodness-of-fit χ^2^ test. Linkage disequilibrium (LD) between pairs of biallelic loci was determined using two measures, *D*’ and *r* ^2^. Unconditional logistic regression analysis was used to calculate odds ratios (ORs) and 95% confidence intervals (CIs), with adjustment for possible confounders (age as a scale variable, sex as a nominal variable and *H*. *pylori* antibody as a nominal variable). Survival functions of the gastric cancer patients within each SNP were plotted by Kaplan-Meier method and compared by Log-Rank test. Hazard ratios (HR) with 95% CIs were used to quantify the influence of genotypes of each SNP on overall survival and were calculated with Cox regression model. For haplotypes with frequencies >1%, risks on susceptibility and death hazard were evaluated comparing to the reference haplotype (major haplotype in the control group) using the unconditional logistic regression model and Cox regression model respectively with the THESIAS version 3.1 software [27, 28]. All statistical tests were two-tailed and *P* value <0.05 was considered to be statistically significant. Unless otherwise stated, analyses above were performed in SAS 9.1.3 software (SAS Institute Inc, USA).

## Results

### Subject characteristics

A total of 1519 subjects, 447 gastric cancer cases, 111 gastric atrophy cases and 961 tumor-free controls were included in this study. The characteristics of all subjects are summarized in Table 1. The demographical factors, age and gender, were not uniformly distributed in the three groups that the GC group had more male and older subjects. And 69.1% of the GC cases were positive for *H*.*pylori*, significantly higher than the control group (49.7%, *P*<0.001) whereas non-significantly lower than the gastric atrophy group (75.7%, *P* = 0.176). As these factors may confound the effects of SNPs, comparisons of genotype distribution below were adjusted by age, sex and *H*.*pylori* infection. Most gastric cancer cases had intestinal type of cancer (87.3%) and 140 patients received postoperative chemotherapy (31.3%).

**Table 1:** Characteristics of the subjects.

Characteristics	Controls, n (%)	GA, n (%)	GC, n (%)	*P* value
N	961	111	447	
Sex				
Male	564(58.7)	66(59.5)	322(72.0)	<0.001
Female	397(41.3)	45(40.5)	125(28.0)	
Age				
≤45	285(29.7)	20(18.0)	36(8.0)	<0.001
46–65	607(63.2)	81(73.0)	239(53.5)	
>65	69(7.2)	10(9.0)	172(38.5)	
*H*.*pylori* antibody				
Negative	483(50.3)	27(24.3)	138(30.9)	<0.001
Postive	478(49.7)	84(75.7)	309(69.1)	
Tumor Sizes				
<5cm			236(52.8)	
≥5cm			191(42.7)	
Undetermined			20(4.5)	
Differentiation				
Moderate to well			183(40.9)	
Poor			252(56.4)	
Undetermined			12(2.7)	
Lauren classfication				
Diffuse			33(7.4)	
Intestinal			390(87.3)	
Mixed			23(5.1)	
Undetermined			1(0.2)	
TNM stage				
I-II			248(55.5)	
III-IV			198(44.3)	
Undetermined			1(0.2)	
Chemotherapy				
XELOX^a^			30(6.7)	
FOLFOX-4^b^			78(17.4)	
Others^c^			32(7.2)	
None			307(68.7)	

TNM: Tumor, Lymph Node, Metastasis.

^a^FOLFOX-4 (5-fluorouracil, leucovorin and oxaliplatin).

^b^XELOX (capecitabine and oxaliplatin).

^c^Other chemotherapies included: 5-fluorouracil; xeloda alone; paclitaxel plus leucovorin and tegafurum; LV5-FU2 (leucovorin plus 5-fluorouracil); FOLFIRI (irinotecan, 5-fluorouracil and leucovorin).

### Association of SNPs with risk of gastric cancer or gastric atrophy

Four SNP loci, rs1569686, rs4911107, rs4911259 and rs8118663, were consistent with the Hardy-Weinberg equilibrium in the control group (*P* was 0.321, 0.341, 0.362 and 0.874, respectively). The rs6119954 locus, however, was found to be deviated from it (*P* = 0.0331). Nonetheless, this locus was included into the final analysis since the concordance rate of the duplicate samples of rs6119954 was 100%. Pair-wise measurements of linkage disequilibrium were listed in S1 Table. Three loci, rs1569686, rs4911107 and rs4911259 showed complete linkage disequilibrium (*D*’>0.98, *r* ^2^>0.96). Therefore, only rs1569686 were analyzed representing rs4911107 and rs4911259.

Comparing to the most common genotype of each SNP, no difference was observed on the distributions of the three loci between gastric cancer group and the control group after adjusting for age, sex and *H*.*pylori* infection (Table 2). None allele or haplotype was associated with risk of gastric cancer. Similar negative results were obtained on the risk of gastric atrophy (Table 2). Moreover, no associations were observed between SNPs or haplotype and risk of *H*.*pylori* infection in the control group (S3 Table).

**Table 2:** ORs and 95% CIs of *DNMT3b* polymorphisms for gastric atrophy and gastric cancer.

Genotype	Controls(%) n = 961	GC(%) n = 447	OR(95%CI)^a^	*P* value	GA(%) n = 111	OR(95%CI)^a^	*P* value
rs6119954							
GG	45.1	44.8	Reference		52.3	Reference	
GA	46.3	44.6	1.00(0.76–1.31)	1.00	38.7	0.70(0.46–1.07)	0.10
AA	8.6	10.6	1.37(0.88–2.13)	0.17	9.0	0.89(0.43–1.83)	0.75
rs1569686							
TT	83.4	80.5	Reference		82.9	Reference	
TG	15.6	18.3	1.14(0.82–1.60)	0.44	16.2	0.99(0.58–1.70)	0.97
GG	1.0	1.1	1.33(0.41–4.31)	0.64	0.9	0.92(0.11–7.56)	0.94
rs4911107							
AA	83.2	80.5	Reference		82.9	Reference	
AG	15.7	18.3	0.86(0.26–2.88)	0.81	16.2	1.07(0.12–9.14)	0.95
GG	1.1	1.1	0.76(0.23–2.46)	0.64	0.9	1.08(0.13–8.86)	0.94
rs4911259							
GG	83.1	80.3	Reference		82.9	Reference	
GT	15.8	18.6	0.86(0.26–2.89)	0.81	16.2	1.06(0.12–9.04)	0.96
TT	1.1	1.1	0.76(0.23–2.45)	0.64	0.9	1.09(0.13–8.88)	0.94
rs8118663							
AA	32.7	28.2	Reference		34.2	Reference	
AG	49.2	52.1	1.28(0.95–1.72)	0.10	50.5	0.96(0.62–1.50)	0.96
GG	18.1	19.7	1.32(0.91–1.91)	0.15	15.3	0.76(0.41–1.39)	0.37
Haplotype^b^							
GTA	56.5	54.0	Reference		59.4	Reference	
ATG	31.1	32.5	1.16(0.93–1.44)	0.18	28.4	0.88(0.63–1.21)	0.43
GGG	8.8	10.4	1.25(0.90–1.72)	0.18	9.0	0.96(0.58–1.60)	0.88
GTG	2.9	2.7	1.04(0.57–1.89)	0.90	3.2	1.00(0.42–2.37)	1.00

^a^ORs for each genotype and haplotype were calculated adjusting for age, sex and *H*.*pylori* infection in logistic regression model.

^b^The haplotype was lined with rs6119954, rs1569686 and rs8118663 and displayed as percentage.

### Association of SNPs with clinicopathologic parameters of gastric cancer

Genotypic distributions of SNPs were analyzed according to clinicopathologic parameters such as tumor size, stage, grade and distant metastasis in gastric cancer cases. Patients carrying the AA genotype of rs6119954 had higher frequency of distant metastasis (25.6% *vs*. 9.1%, *P* = 0.003). And patients bearing the AA genotype of rs8118663 had more proportion of tumor size ≥5 cm (33.5% *vs*. 23.3%, *P* = 0.040) (S4 Table).

### Association of SNPs with survival of gastric cancer

Follow-up information was available for 435 (97.3%) gastric cancer patients until February 2015. Thirteen patients died of postoperative complications within 30 days at the beginning of the study and these cases were excluded from the analysis of effects of SNPs on survival. Finally, 422 cases were included in the survival analysis. During the follow-up, 195 (46.2%) patients died from gastric cancer, 14 (3.3%) cases died of other causes, 203 patients (48.1%) lived and 10 (2.4%) patients were lost to follow up. The median follow-up time was 55.1 months (ranging from 1.1 to 79.2 months) for all cases included in the survival analysis.

**Fig 1:**
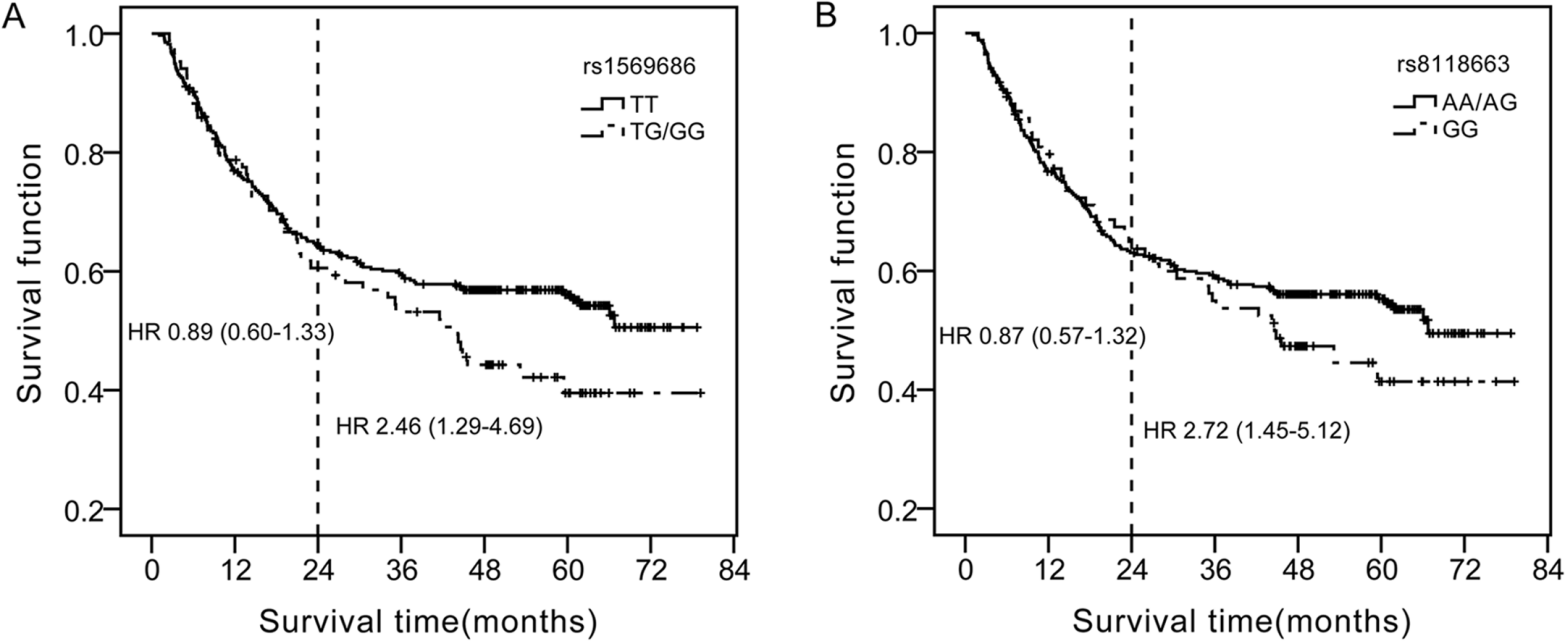
Survival plots of gastric cancer patients. (A) plot for rs1569686 using the dominant model (TG/GG *vs*.TT). Patients carrying rs1569686 TG/GG genotype tended to live shorter than those carrying TT genotype. This trend was more evident in patients who lived longer than 2.0 years with a hazard ratio (HR) 2.46 (95% CI 1.29–4.69) after adjusting for age, sex TNM stage and chemotherapy type. (B) plot for rs8118663 using the recessive model (GG vs. AA/AG). Similar to rs1569686, rs8118663 GG carriers tended to have shorter survival time.

Cox regression analyses was performed to evaluate the association of *DNMT3b* genotypes on survival of gastric cancer using the dominant or recessive model. We found that rs1569686 TG/GG variant genotype was associated with a statistically significant 43% increased risk of death compared to the TT genotype in the dominant model. Moreover, tendency of effects on long-term survival could be observed from the survival plots. Therefore, subgroup analyses were performed in patients living longer than two years. Patients bearing G allele (TG or GG genotype) of 1569686 was found to live shorter than those of TT genotype (HR = 2.46, 95%CI: 1.29–4.69, *P* = 0.006) after adjusting for age, sex, TNM stage and chemotherapy after tumorectomy (Fig 1A). Similar results were observed for the G allele carriers of rs4911107 and T allele carriers of rs4911259 in the overall survival of gastric cancer (Table 3). And those carrying GG genotype of rs8118663 tended to have shorter survival time than A allele carriers (AA or AG) after adjusting for age, sex, TNM stage and chemotherapy after tumorectomy (HR = 2.72, 95%CI: 1.45–5.12, *P* = 0.002) (Fig 1B).

**Table 3:** Association between *DNMT3b* polymorphisms and OS of gastric cancer.

Genotype	All cases			HR (95%CI)^a^	*P* ^a^
	Patients	Deaths	MST (months)		
rs6119954					
GG	189	89	66.07	Reference	
GA	187	87	61.80	0.96(0.71–1.30)	0.771
AA	43	18	50.19*	0.84(0.51–1.40)	0.503
GA+AA *vs*.GG	230	105	49.83	0.93(0.70–1.24)	0.631
rs1569686					
TT	337	147	50.02*	Reference	
TG	80	46	42.30	1.41(1.01–1.98)	0.046
GG	5	2	37.40*	1.99(0.49–8.10)	0.339
TG+GG *vs*.TT	85	48	44.17	1.43(1.02–1.99)	0.036
rs4911107					
AA	337	147	50.02*	Reference	
AG	80	46	42.30	1.41(1.01–1.98)	0.046
GG	5	2	37.40*	1.99(0.49–8.10)	0.339
AG+GG *vs*.AA	85	48	44.17	1.43(1.02–1.99)	0.036
rs4911259					
GG	336	146	50.11*	Reference	
GT	81	47	42.30	1.40(1.00–1.96)	0.047
TT	5	2	37.40*	1.99(0.49–8.11)	0.338
GT+TT *vs*.GG	86	49	44.17	1.42(1.02–1.97)	0.037
rs8118663					
AA	120	55	66.07	Reference	
AG	218	95	50.46	0.84(0.60–1.19)	0.330
GG	84	45	44.60*	1.16(0.78–1.74)	0.466
AA+AG	338	150	66.77	Reference	
GG	84	45	44.60	1.30(0.93–1.82)	0.128
Haplotype^b^					
GTA	-	-	-	Reference	
ATG	-	-	-	0.99(0.79–1.23)	0.913
GGG	-	-	-	1.28(0.93–1.77)	0.132
GTG	-	-	-	0.92(0.48–1.76)	0.791

MST: median survival time. HR: hazard ratio.

*Mean OS was presented when median OS could not be calculated.

^a^HRs and *P* values for each genotype and haplotype were calculated adjusting for age, sex, TNM stage and chemotherapy type using Cox regression model.

^b^The haplotype was lined with rs6119954, rs1569686 and rs8118663.

### Association between SNPs and expression of DNMT3b

DNMT3b expression was evaluated in cancerous tissue of 104 gastric cancer cases using immunohistochemical method. DNMT3b expression was observed mainly in cell nucleus and levels of expression were high (HSCORE>200) in 64 cases (61.5%), moderate (100<HSCORE≤200) in 30 (28.8%), low (HSCORE≤100) in 2 (1.9%) and negative (HSCORE = 0) in 8 (7.7%). Results of expression according to genotypes of the three SNP loci were shown in Table 4. However, no difference of DNMT3b expression could be observed among genotypes of each SNP.

**Table 4:** Influence of DNMT3b polymorphisms on expression of DNMT3b.

Genotype	Intensity of immunostaining n(%)				Median HSCORE (quartile)	*P* value
	0	1+	2+	3+		
rs6119954						
GG(n = 87)	5(5.7)	2(2.3)	24(27.6)	56(64.4)	180(120–240)	0.43
GA(n = 13)	2(15.4)	0	5(38.5)	6(46.2)	160(100–180)	
AA(n = 4)	1(25.0)	0	1(25.0)	2(50.0)	135(23–248)	
rs1569686						
TT(n = 84)	6(7.1)	2(2.4)	24(28.6)	52(61.9)	180(100–240)	0.98
TG/GG(n = 19/1)	2(10.0)	0	6(30.0)	12(60.0)	170(120–240)	
rs8118663						
AA(n = 67)	3(4.5)	2(3.0)	20(29.9)	42(62.7)	180(100–240)	0.10
AG(n = 19)	2(10.5)	0	4(21.1)	13(68.4)	210(140–240)	
GG(n = 18)	3(16.7)	0	6(33.3)	9(50.0)	150(80–180)	

## Discussion

In the present study, we systematically explored the role of variants of *DNMT3b* gene in the development and prognosis of gastric cancer in a large population. We found that rs1569686 TG/GG genotypes were significantly associated with poor survival of gastric cancer. Furthermore, we observed that *DNMT3b* polymorphisms affect long-term survival of gastric cancer and the effect was even stronger in patients living longer than two years.

Most works published only focus on the role of *DNMT3b* in the carcinogensis of various malignancies. As far as we know, this is the first report describing the association between *DNMT3b* polymorphism and gastric cancer survival in a Chinese population. Our current study showed that *DNMT3b* variants contributed to the long-term survival of gastric cancer. The *DNMT3b* rs1569686 variant TG or GG genotype carriers showed increased HR with a *P*-value of 0.036 (HR = 1.43, 95CI:1.02–1.99) when compared with TT wild-type. Besides, individual bearing the G allele of rs1569686 (TG or GG) have 2.46-fold increase of mortality risk than non G allele carriers in patients living longer than 2 years (Fig 1A), and individuals carrying rs8118663 GG genotype tend to have higher risk in the long-term survival (Fig 1B).

We further for the first time investigated the association between *DNMT3b* polymorphisms and clinical characteristics of gastric cancer patients. Patients carrying the rs6119954 AA genotype had more frequency of distant metastasis, suggesting that rs6119954 polymorphism may be involved in the progression of gastric cancer. Similarly, patients with rs8118663 AA genotype tended to have larger tumor size (≥5cm), suggesting that rs8118663 SNP may play a role in the tumor progression of GC patients. Taken together, all these findings demonstrated the predictive value of *DNMT3b* polymorphisms for the progression and survival prognosis in patients with gastric cancer.

The *DNMT3b* gene encodes DNMT3b, a family member of DNA methyltransferase that participates in a wide range of biological processes, including tumor development and tumorgenesis [29]. Several previous studies have shown that polymorphisms of *DNMT3b* are associated with cancer development in a variety of tumors. Shen et al [18] firstly reported that the carriers of T alleles, particularly heterozygous (CT) of rs2424913, had nearly 2-fold increased risk of lung cancer risk compared to the homozygous CC genotype in Caucasian population. In addition, this C-to-T common genetic variant in the DNMT3b promoter was found to profoundly increase mRNA expression [30]. However, the role of rs2424913 on cancer in Chinese could be negligible as the C allele in Chinese population is absent or rare [20], the significance of great diversity in *DNMT3b* SNP distribution may be due to different ethnic groups, unknown environmental factors or the interplay between environmental factors and genetic predisposition. The rs1569686 was reported to be associated with the susceptibility of lung cancer [31] and gastric cancer [21] but not in hepatocellular carcinoma [19], esophagus cancer [32] and nasopharyngeal carcinoma [20]. In a previous meta-analysis based on 24 case-control studies, the *DNMT3b* rs1569686 G allele has been identified as a low risk factor for developing colorectal cancer [33]. The rs1569686 is located in the CpG poor promoter region. In vitro promoter assay have revealed that rs1569686 polymorphism does not affect the transcriptional activity of the *DNMT* promoter [34], but the exact mechanisms are largely unknown. A study by Lee et al [35] showed that the rs6087990 in the promoter region of *DNMT3b*, which was in complete LD with rs1569686, could modify the promoter activity of *DNMT3b*, the transcription activity of the T allele was significantly lower compared with the C allele, but the rs1569686 polymorphism did not affect the promoter activity. In our study, however, we did not observe any association of rs1569686 with the development of gastric cancer or gastric atrophy, an important risk factor for gastric cancer (Table 2). Also, we did not observe any differences of DNMT3b expression within the genotypes of each SNPs using immunohistochemistry method. The underlying mechanism of *DNMT3b* on gastric cancer needs to be clarified in future studies. Another sites, rs2424908, which is in complete linkage disequilibrium with rs8118663 (*r* ^2^ = 0.956), has also been found to be involved in tumor, such as oesophagus cancer [32] and colorectal cancer [34]. However, in our study, no positive association with gastric cancer could be observed. Similar to our findings, Yang et al reported that *DNMT3b* rs2424908 polymorphisms were not associated with the susceptibility to gastric cancer in the southern Chinese population [36]. Therefore, more studies may be needed to conclude the role of genetic polymorphism of *DNMT3b* in the development of gastric cancer.

Two limitations should be acknowledged in our study. One was that only SNPs with a minimum minor allele of 0.10 or greater were included due to the selection criteria. Low frequency variations may also correlate with cancer development such as rs2424913 [22]. Further study covering the SNPs with low frequency is needed. The other one is that the follow-up time for gastric cancer cases seems insufficient as most of cases on the right side of the survival plots are censored. Influences of SNPs on long-term survival could not be fully addressed though a trend could be observed. However, when the cases are stratified into subgroup living longer than two years, a significant association on long-term prognosis could be observed. Nonetheless, role of *DNMT3b* on gastric cancer will be revaluated and updated in future as the follow-up is still ongoing.

## Conclusions

In summary, we find polymorphisms of *DNMT3b* may affect the overall survival of gastric cancer, suggesting the potential of *DNMT3b* SNPs as a useful marker to predict overall survival of gastric cancer, especially in patients surviving longer than two years. However, we did not observe any significant associations between *DNMT3b* variants and risk of gastric cancer or gastric atrophy. Further investigations are needed to fully clarify the role of the *DNMT3b* on gastric cancer in different ethnic populations.

## Supporting Information

S1 Table Linkage disequilibrium coefficients (*D*’) and *r* ^2^ between *DNMT3b* htSNPs. (DOCX)

S2 Table Clinical data of gastric cancer patients. (DOCX)

S3 Table Association of SNPs and *H*.*pylori* infection in the control group. (DOCX)

S4 Table Distributions of genotypes according to clinical parameters in gastric cancer cases. (DOCX)

S1 Supporting Data The clinical data and genotype frequencies of DNMT3b gene polymorphisms in cases and controls. (XLS)
