# Supplementary material for: Polymorphism of DNA Methyltransferase 3b and Association with Development and Prognosis in Gastric Cancer
Source: PLoS One. 2015 Aug 11;10(8):e0134059. doi: 10.1371/journal.pone.0134059 (PMC4532499; doi:10.1371/journal.pone.0134059)
Supplement: S1 Table — (DOCX) [file pone.0134059.s001.docx]

**S1Table. Linkage disequilibrium coefficients (*D*’) and *r*^2^ between *DNMT3b* htSNPs**

|  | | *D*’ | | | | |
| --- | --- | --- | --- | --- | --- | --- |
|  |  | rs6119954 G>A | rs1569686 T>G | rs4911107 A>G | rs4911259 G>T | rs8118663 A>G |
| *r*^2^ | rs6119954 G>A | - | 0.7301 | 0.9458 | 0.9468 | 0.0170 |
|  | rs1569686 T>G | 0.1592 | - | 0.9935 | 0.9871 | 0.1939  . |
|  | rs4911107 A>G | 0.2018 | 0.9719 | - | 1.0000 | 0.2182 |
|  | rs4911259 G>T | 0.20260 | 0.9687 | 0.9968 | - | 0.2074 |
|  | rs8118663 A>G | 0.0100 | 0.0717 | 0.0790 | 0.0753 | - |
