# Supplementary material for: Polymorphism of DNA Methyltransferase 3b and Association with Development and Prognosis in Gastric Cancer
Source: PLoS One. 2015 Aug 11;10(8):e0134059. doi: 10.1371/journal.pone.0134059 (PMC4532499; doi:10.1371/journal.pone.0134059)
Supplement: S2 Table — (DOCX) [file pone.0134059.s002.docx]

**S2 Table. Clinical data of gastric cancer patients**

| Variables | Patients N(%) | Deaths N(%) | MST | HR (95% CI) | Log-rank *P* |
| --- | --- | --- | --- | --- | --- |
| Age |  |  |  |  |  |
| <60 | 196(46.4) | 86(44.1) | 66.77 | Reference | 0.377 |
| ≥60 | 226(53.6) | 109(55.9) | 61.80 | 1.14(0.86-1.51) |  |
| Gender |  |  |  |  |  |
| Male | 305(72.3) | 151(77.4) | 46.98* | Reference | 0.041 |
| Female | 117(27.7) | 44(22.6) | 54.25 | 0.71(0.51-0.99) |  |
| Tumor Sizes |  |  |  |  |  |
| <5cm | 231(55.8) | 76(40.2) | 58.90* | Reference | <0.001 |
| ≥5cm | 183(44.2) | 113(59.8) | 37.68 | 2.52(1.89-3.38) |  |
| TMN Stages |  |  |  |  |  |
| I | 66(15.6) | 5(2.6) | 72.53* | Reference | <0.001 |
| II | 174(41.3) | 51(26.2) | 61.61* | 4.28(1.71-10.73) |  |
| III | 152(36.0) | 116(59.4) | 27.94 | 19.66(8.02-48.23) |  |
| IV | 30(7.1) | 23(11.8) | 23.84 | 21.61(8.20-56.95) |  |
| Tumor differentiation |  |  |  |  |  |
| Well | 155(37.1) | 69(35.9) | 66.77 | Reference | 0.160 |
| Moderate/poor | 263(62.9) | 123(64.1) | 47.30* | 1.24(0.92-1.66) |  |
| Lauren classfication |  |  |  |  |  |
| Diffuse | 31(7.4) | 19(9.7) | 28.20 | Reference | 0.140 |
| Intestinal | 368(87.4) | 168(86.2) | 49.59* | 0.65(0.40-1.05) |  |
| Mixed | 22(5.2) | 8(4.1) | 59.43 | 0.50(0.22-1.15) |  |
| Depth of invasion |  |  |  |  |  |
| T1 | 43(10.3) | 4(2.1) | 71.97* | Reference | <0.001 |
| T2 | 54(12.9) | 8(4.1) | 70.29* | 1.63(0.49-5.40) |  |
| T3 | 303(72.3) | 165(85.5) | 36.17 | 8.26(3.06-22.29) |  |
| T4 | 19(4.5) | 16(8.3) | 11.40 | 19.82(6.61-59.42) |  |
| Lymph node metastasis |  |  |  |  |  |
| N0 | 119(28.4) | 14(7.3) | 70.23* | Reference | <0.001 |
| N1 | 122(29.1) | 46(23.8) | 56.20* | 3.74(2.05-6.80) |  |
| N2 | 91(21.7) | 58(30.1) | 23.60 | 8.50(4.73-15.26) |  |
| N3 | 87(20.8) | 75(38.8) | 9.83 | 19.65(11.03-35.02) |  |
| Distant metastasis |  |  |  |  |  |
| M0 | 392(92.9) | 172(88.2) | 50.81* | Reference | <0.001 |
| M1 | 30(7.1) | 23(11.8) | 13.27 | 2.75(1.78-4.26) |  |
| Chemotherapy |  |  |  |  |  |
| XELOX^a^ | 29(6.9) | 9(4.6) | 54.75* | Reference | 0.196 |
| FOLFOX-4^b^ | 77(18.2) | 36(18.5) | 45.70* | 1.74(0.84-3.60) |  |
| Others^c^ | 32(7.6) | 20(10.3) | 38.41 | 2.35(1.07-5.16) |  |
| None | 284(67.3) | 130(66.7) | 49.10 | 1.73(0.88-3.39) |  |

MST: median survival time. HR: hazard ratio.

*Mean OS was presented when median OS could not be calculated.

^a^FOLFOX-4 (5-fluorouracil, leucovorin and oxaliplatin).

^b^XELOX (capecitabine and oxaliplatin).

^c^Other chemotherapies included: 5-fluorouracil; xeloda alone; paclitaxel plus leucovorin and tegafurum; LV5-FU2 (leucovorin plus 5-fluorouracil); FOLFIRI (irinotecan ,5-fluorouracil and leucovorin).
