# Supplementary material for: Polymorphism of DNA Methyltransferase 3b and Association with Development and Prognosis in Gastric Cancer
Source: PLoS One. 2015 Aug 11;10(8):e0134059. doi: 10.1371/journal.pone.0134059 (PMC4532499; doi:10.1371/journal.pone.0134059)
Supplement: S3 Table — (DOCX) [file pone.0134059.s003.docx]

**S3 Table. Relationship of *H.pylori* infection and SNPs in the control group**

| Genotype | *H.pylori*^+^(%) N=478 | *H.pylori*^-^(%) N=483 | OR(95%CI)^a^ | *P* value |
| --- | --- | --- | --- | --- |
| rs6119954 |  |  |  |  |
| GG | 44.1 | 46.0 | Reference |  |
| GA | 47.1 | 45.5 | 1.06(0.82-1.39) | 0.65 |
| AA | 8.8 | 8.5 | 1.07(0.67-1.71) | 0.79 |
| rs1569686 |  |  |  |  |
| TT | 82.0 | 84.7 | Reference |  |
| TG | 16.9 | 14.3 | 1.22(0.86-1.73) | 0.28 |
| GG | 1.0 | 1.0 | 1.01(0.29-3.51) | 0.99 |
| rs4911107 |  |  |  |  |
| AA | 82.0 | 84.5 | Reference |  |
| AG | 16.9 | 14.5 | 1.00(0.29-3.50) | 0.99 |
| GG | 1.0 | 1.0 | 1.20(0.84-1.70) | 0.32 |
| rs4911259 |  |  |  |  |
| GG | 81.8 | 84.5 | Reference |  |
| GT | 17.2 | 14.5 | 1.01(0.29-3.51) | 0.99 |
| TT | 1.0 | 1.0 | 1.21(0.86-1.72) | 0.28 |
| rs8118663 |  |  |  |  |
| AA | 31.0 | 34.2 | Reference |  |
| AG | 49.8 | 48.7 | 1.12(0.84-1.50) | 0.42 |
| GG | 19.2 | 17.2 | 1.22(0.84-1.79) | 0.30 |
| Haplotype^b^ |  |  |  |  |
| GTA | 55.1 | 57.8 | Reference |  |
| ATG | 31.6 | 30.6 | 1.08(0.88-1.33) | 0.44 |
| GGG | 9.5 | 8.2 | 1.21(0.88-1.66) | 0.24 |
| GTG | 3.1 | 2.7 | 1.20(0.70-2.06) | 0.50 |

^a^ORs for each genotype and haplotype were calculated adjusting for age and sex in logistic regression model.

^b^The haplotype was lined with rs6119954, rs1569686 and rs8118663 and displayed as percentage.
