# Supplementary material for: Polymorphism of DNA Methyltransferase 3b and Association with Development and Prognosis in Gastric Cancer
Source: PLoS One. 2015 Aug 11;10(8):e0134059. doi: 10.1371/journal.pone.0134059 (PMC4532499; doi:10.1371/journal.pone.0134059)
Supplement: S4 Table — (DOCX) [file pone.0134059.s004.docx]

**S4 Table. Distributions of genotypes according to clinical parameters in gastric cancer cases**

|  | rs6119954 | | | |  | rs1569686 | | | |  | rs8118663 | | | |
| --- | --- | --- | --- | --- | --- | --- | --- | --- | --- | --- | --- | --- | --- | --- |
|  | GG | GA | AA | *P* |  | TT | TG | GG | *P* |  | AA | AG | GG | *P* |
| Age | 62(54-70) | 60(53-70) | 62 (52-72) | 0.722 |  | 61(54-70) | 62(54-71) | 51(47-66) | 0.415 |  | 62(55-70) | 60(53-71) | 62(53-70) | 0.866 |
| Sex |  |  |  |  |  |  |  |  |  |  |  |  |  |  |
| Male | 45.5 | 43.3 | 11.3 | 0.583 |  | 81.1 | 18.0 | 0.9 | 0.716 |  | 28.3 | 52.8 | 18.9 | 0.809 |
| Female | 43.2 | 48.0 | 8.8 |  |  | 79.2 | 19.2 | 1.6 |  |  | 28.0 | 50.4 | 21.6 |  |
| *H.pylori* |  |  |  |  |  |  |  |  |  |  |  |  |  |  |
| Negative | 39.9 | 47.8 | 12.3 | 0.343 |  | 84.1 | 15.2 | 0.7 | 0.524 |  | 26.8 | 52.2 | 21.0 | 0.855 |
| Positive | 47.1 | 43.1 | 9.8 |  |  | 79.0 | 19.7 | 1.3 |  |  | 28.8 | 52.1 | 19.1 |  |
| Tumor size |  |  |  |  |  |  |  |  |  |  |  |  |  |  |
| <5cm | 41.3 | 48.9 | 9.8 | 0.182 |  | 78.4 | 20.8 | 0.8 | 0.346 |  | 23.3 | 57.6 | 19.1 | 0.040 |
| ≥5cm | 48.9 | 40.0 | 11.1 |  |  | 82.7 | 15.7 | 1.6 |  |  | 33.5 | 46.6 | 19.9 |  |
| Differentiation |  |  |  |  |  |  |  |  |  |  |  |  |  |  |
| Poor | 45.6 | 42.1 | 12.3 | 0.194 |  | 81.0 | 17.1 | 2.0 | 0.124 |  | 27.8 | 52.0 | 20.2 | 0.942 |
| Moderate to well | 43.3 | 48.9 | 7.8 |  |  | 79.8 | 20.2 | 0 |  |  | 29.0 | 51.9 | 19.1 |  |
| Lauren classfication |  |  |  |  |  |  |  |  |  |  |  |  |  |  |
| Diffuse | 45.5 | 36.4 | 18.2 | 0.124 |  | 84.8 | 12.1 | 3.0 | 0.413 |  | 27.3 | 51.5 | 21.2 | 0.743 |
| Intestinal | 44.4 | 46.3 | 9.3 |  |  | 80.8 | 18.2 | 1.0 |  |  | 28.7 | 52.3 | 19.0 |  |
| Mixed | 47.8 | 30.4 | 21.7 |  |  | 73.9 | 26.1 | 0 |  |  | 21.7 | 47.8 | 30.4 |  |
| TNM stage |  |  |  |  |  |  |  |  |  |  |  |  |  |  |
| I-II | 44.7 | 45.5 | 9.8 | 0.769 |  | 78.6 | 20.2 | 1.2 | 0.524 |  | 28.2 | 51.6 | 20.2 | 0.966 |
| III-IV | 45.2 | 43.1 | 11.7 |  |  | 82.8 | 16.2 | 1.0 |  |  | 28.3 | 52.5 | 19.2 |  |
| Distant metastasis |  |  |  |  |  |  |  |  |  |  |  |  |  |  |
| M0 | 44.4 | 46.4 | 9.1 | 0.003 |  | 80.1 | 18.6 | 1.2 | 0.892 |  | 27.9 | 52.5 | 19.6 | 0.900 |
| M1 | 48.7 | 25.6 | 25.6 |  |  | 84.6 | 15.4 | 0 |  |  | 30.8 | 48.7 | 20.5 |  |
| Chemotherapy |  |  |  |  |  |  |  |  |  |  |  |  |  |  |
| XELOX | 53.3 | 40.0 | 6.7 | 0.597 |  | 80.0 | 20.0 | 0 | 0.656 |  | 36.7 | 43.3 | 20.0 | 0.592 |
| FOLFOX-4 | 47.7 | 39.5 | 6.3 |  |  | 83.3 | 14.1 | 2.6 |  |  | 32.1 | 47.4 | 20.5 |  |
| Others | 34.4 | 59.4 | 6.3 |  |  | 75.0 | 25.0 | 0 |  |  | 15.6 | 62.5 | 21.9 |  |
| None | 44.4 | 44.8 | 10.8 |  |  | 80.5 | 18.6 | 1.0 |  |  | 27.7 | 53.1 | 19.2 |  |
